# Supplementary figures and images for: Preliminary Investigation about Aspergillus spp. Spread in Umbrian Avian Farms
Source: J Fungi (Basel). 2022 Nov 16;8(11):1213. doi: 10.3390/jof8111213 (PMC9698509; doi:10.3390/jof8111213)

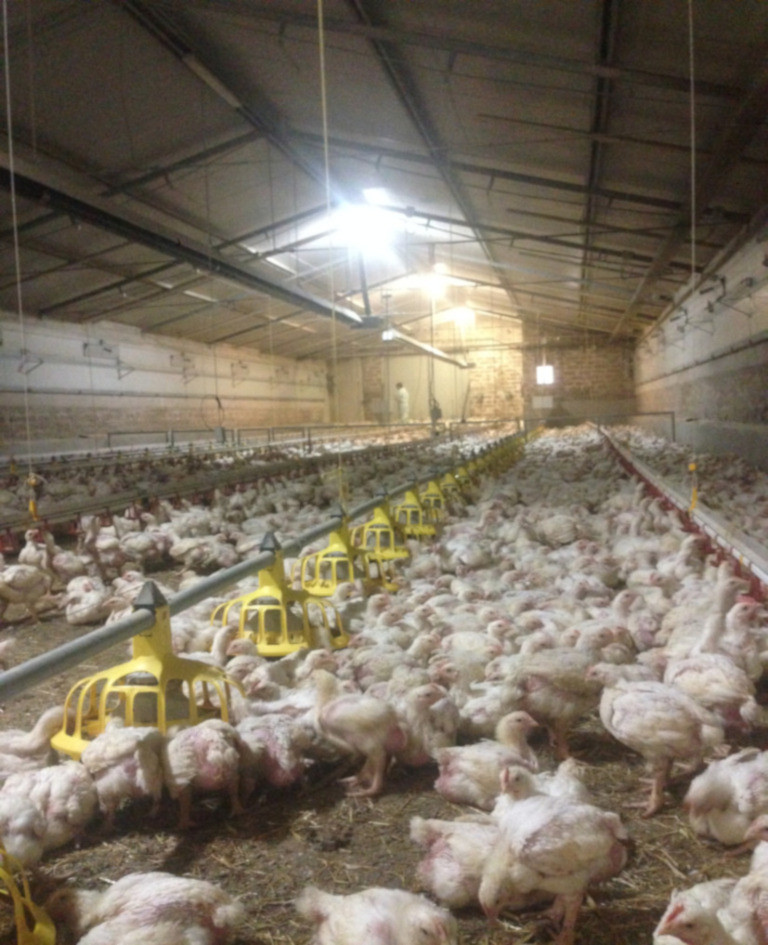

Supplement: Supplementary file 1 [file jof-08-01213-s001.zip › Figure S1.jpeg]

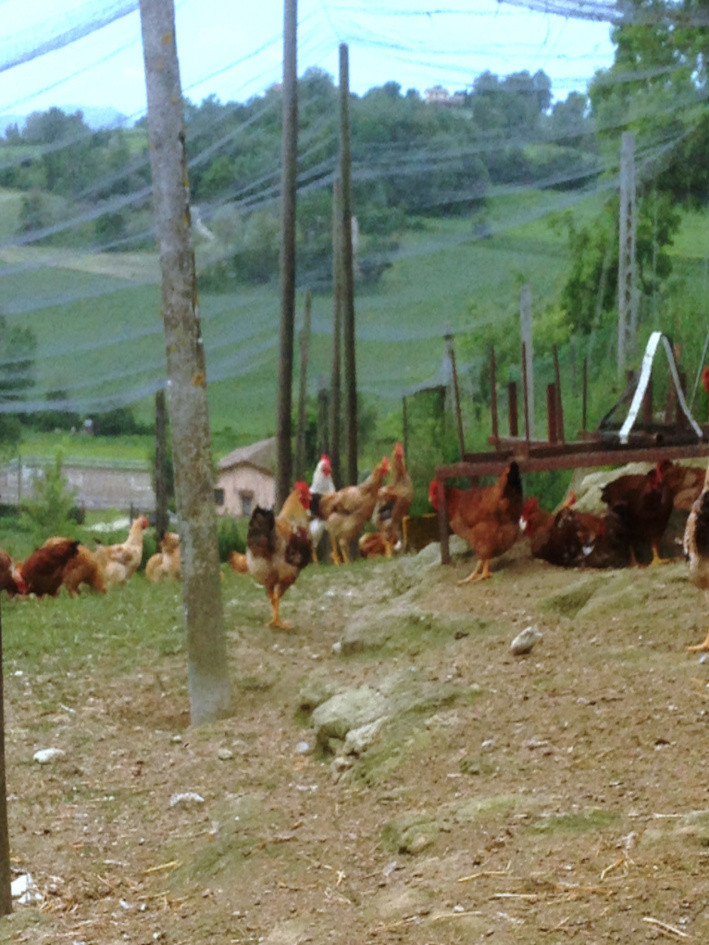

Supplement: Supplementary file 1 [file jof-08-01213-s001.zip › Figure S2.jpeg]
